# Reduced Creatine Kinase B Activity in Multiple Sclerosis Normal Appearing White Matter

**DOI:** 10.1371/journal.pone.0010811

**Published:** 2010-05-25

**Authors:** Christel Steen, Nadine Wilczak, Johannes M. Hoogduin, Marcus Koch, Jacques De Keyser

**Affiliations:** 1 Department of Neurology, University Medical Center Groningen, University of Groningen, Groningen, The Netherlands; 2 Department of Radiology, University Medical Center Utrecht, Utrecht, The Netherlands; 3 BCN-Neuroimaging Center, University Medical Center Groningen, University of Groningen, Groningen, The Netherlands; 4 Department of Neurology, UZ Brussel, Vrije Universiteit Brussel, Brussels, Belgium; Julius-Maximilians-Universität Würzburg, Germany

## Abstract

**Background:**

Two studies using ^31^P-magnetic resonance spectroscopy (MRS) reported enhanced phosphocreatine (PCr) levels in normal appearing white matter (NAWM) of subjects with multiple sclerosis (MS), but this finding could not be properly explained.

**Methodology/Principal Findings:**

We performed ^31^P-MRS and ^1^H-MRS in the NAWM in 36 subjects, including 17 with progressive MS, 9 with benign MS, and 10 healthy controls. Compared to controls, PCr/β-ATP and PCr/total ^31^P ratios were significantly increased in subjects with progressive MS, but not with benign MS. There was no correlation between PCr ratios and the N-acetylaspartate/creatine ratio, suggesting that elevated PCr levels in NAWM were not secondary to axonal loss. In the central nervous system, PCr is degraded by creatine kinase B (CK-B), which in the white matter is confined to astrocytes. In homogenates of NAWM from 10 subjects with progressive MS and 10 controls without central nervous system disease, we measured CK-B levels with an ELISA, and measured its activity with an enzymatic assay kit. Compared to controls, both CK-B levels and activity were decreased in subjects with MS (22.41 versus 46.28 µg/ml; p = 0.0007, and 2.89 versus 7.76 U/l; p<0.0001).

**Conclusions/Significance:**

Our results suggest that PCr metabolism in the NAWM in MS is impaired due to decreased CK-B levels. Our findings raise the possibility that a defective PCr metabolism in astrocytes might contribute to the degeneration of oligodendrocytes and axons in MS.

## Introduction

Relapses and progression are the two basic clinical courses of multiple sclerosis (MS), which is traditionally viewed as a T-cell driven autoimmune disease against myelin. There is substantial evidence that inflammation plays a role in relapses, but whether this is a primary or secondary phenomenon remains a matter of controversy. Pathological studies have shown that early lesions and some evolving focal demyelinating lesions are characterized by oligodendrocyte apoptosis with little or no lymphocytic infiltration [1]–[3]. The progressive phase of MS reflects an insidious axonal degeneration that is age related, and independent of relapses [4]–[5]. The pathophysiological mechanism underlying this diffuse axonal degeneration is unknown. There is little evidence that inflammatory mechanisms play a primary role in this process as immunomodulatory drugs are ineffective in progressive MS. Demyelination and axonal degeneration in MS continue despite pronounced immunosuppression [6].

**Figure 1:**
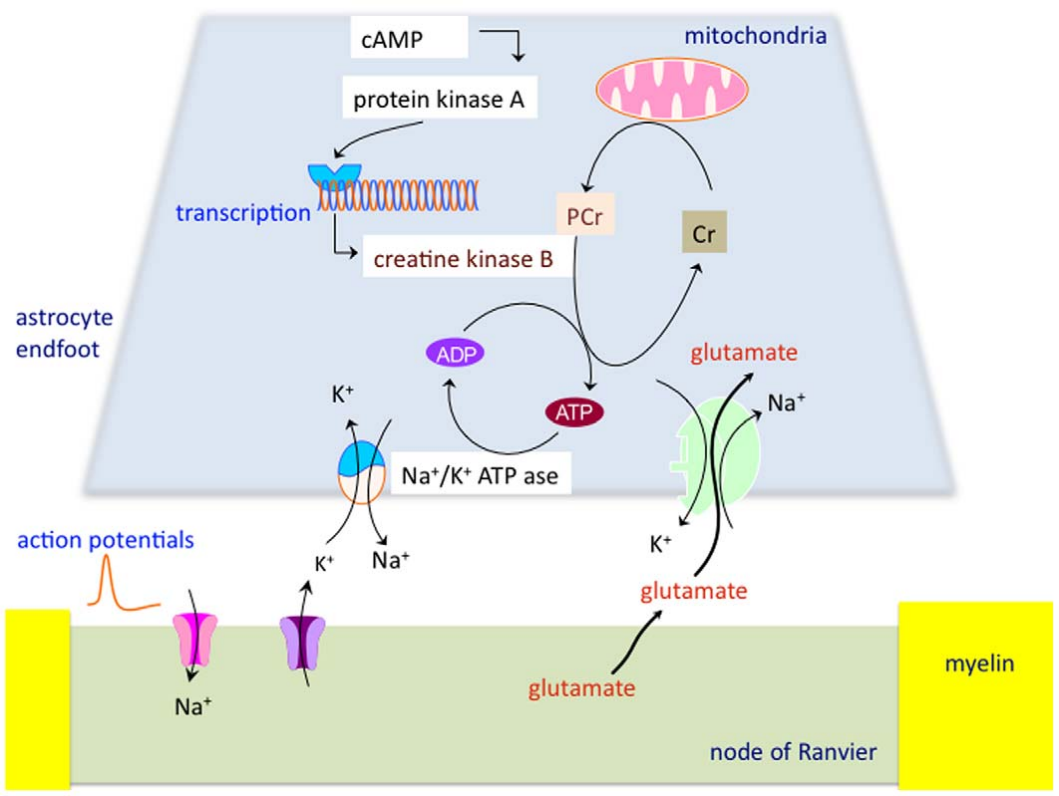
Proposed model of PCr metabolism in astrocytes. PCr generated by mitochondria diffuses in the astrocytic processes where it is degraded by CK-B to deliver ATP. Free Cr, formed by the removal of phosphate from PCr, diffuses back to the mitochondria. This local energy is required for the Na^+^/K^+^-ATPase, which removes K^+^ generated during axonal electrogenesis from the extracellular space, and establishes the Na^+^ gradient required for Na^+^-dependent glutamate uptake by astrocytes. Transcription of CK-B appears to be mediated by cAMP [23] [24].

Astrocytes in the white matter extend thin processes that contact axons at the nodes of Ranvier, where they are presumed to participate in the regulation of homeostatic and metabolic functions necessary for a proper activity of axons [7]. These astrocytic end-feet are too thin to contain mitochondria [8], and energy metabolism is locally supplied by glycolysis, glycogenolysis and phosphocreatine (PCr) breakdown [9]. PCr, a smaller molecule than adenosine triphosphate (ATP), is generated by mitochondria and diffuses in the astrocytic processes where it is degraded by creatine kinase B (CK-B) to deliver ATP. Free creatine (Cr), formed by the removal of phosphate from PCr, then diffuses back to the mitochondria for rephosphorylation (**Figure 1**). It has been shown by immunohistochemistry that CK-B in both adult human and adult mice white matter is confined to astrocytes [10] [11] [12]. The most ATP consuming activity in astrocytic processes during axonal electrogenesis is the Na^+^/K^+^-ATPase. It takes up K^+^ released by axons in the extracellular space after each depolarization, and it establishes the Na^+^ gradient necessary for glutamate uptake by the astrocytic Na^+^-dependent glutamate transporter (**Figure 1**) [13] [14].

Two previous studies using ^31^phosphorus magnetic resonance spectroscopy (^31^P-MRS), reported that normal appearing white matter (NAWM) PCr/β-ATP and PCr/total ^31^P ratios were higher in MS subjects than in healthy controls [15] [16]. As there was no clear explanation, these findings have received little further attention. In the present study we sought to confirm the previous findings, relate PCr levels to the degree of axonal loss, and assess CK-B activity and levels in NAWM of subjects with MS.

## Materials and Methods

### Subjects

The Ethics Committee of the University Medical Center of Groningen approved the MRS studies, and all subjects provided written informed consent. Thirty-six subjects including 17 with progressive MS (9 primary and 8 secondary), 9 with benign MS, and 10 healthy controls (**Table 1**) were included. Benign MS subjects had Extended Disability Status Scale scores of ≤3 at least 15 years after disease onset. All MS subjects were clinically stable without evidence of an acute exacerbation within the 3 months prior to scanning. Besides one patient with secondary progressive MS who used beta-interferon, no immunomodulatory drugs were used.

**Table 1:** Clinical characteristics of healthy controls and MS subjects in the MRS studies.

	Healthy controls n = 10	Benign MS n = 9	Progressive MS n = 17
Male/female	5/5	5/4	9/8
Age (years), mean ± SD	49.7 (6.3)	51.0 (5.2)	50.5 (7.3)
Disease duration (years), mean ± SD	-	19.1 (5.9)	21.0 (12.0)
Expanded Disability Status Scale (range)	-	1.6 (0–2.5)	5.3 (4.0–6.5)

### ^31^P MRS and ^1^H MRS

**Figure 2:**
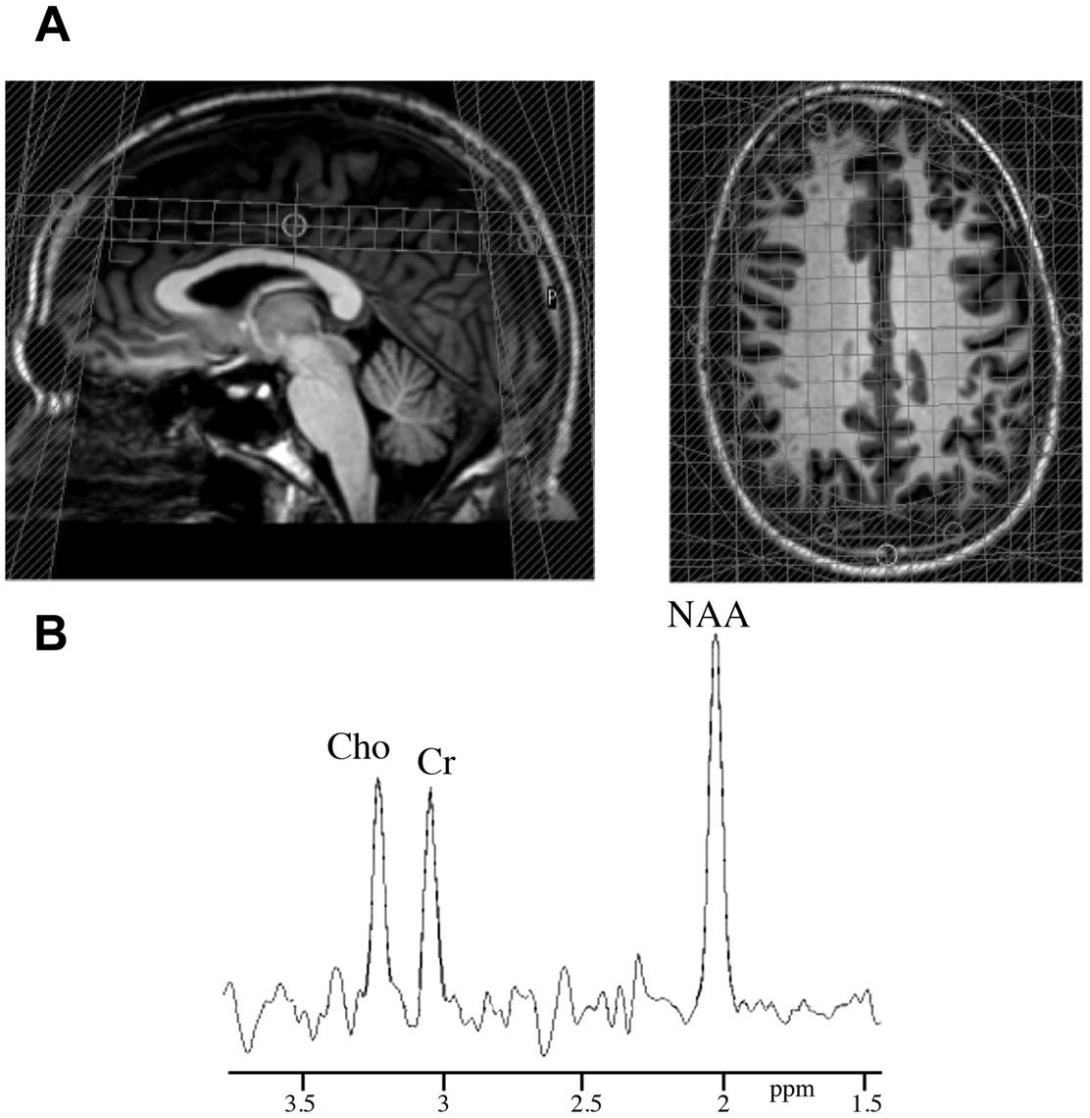
^1^H Magnetic resonance spectroscopy. (**A**) Slab with multiple voxels placed in the centrum semiovale for ^1^H MRS and (**B**) ^1^H-spectrum showing the peaks for choline (Cho), creatine (Cr), and N-acetylaspartate (NAA).

MRI scans of the brain were obtained using a 3 Tesla Philips MRI system with either the 8-channel SENSE head coil for ^1^H MRS, or the phosphorus surface coil (diameter 14 cm) for ^31^P MRS. ^1^H MRS was preceded by the acquisition of a transverse 3D T1-weighted scan, which was reformatted to obtain coronal and sagittal series. This allowed accurate positioning of the 2D spectroscopic imaging slab, containing multiple voxels of 1.5 cm^3^, in the centrum semiovale above the corpus callosum (**Figure 2**). Water suppressed single slice PRESS was used with the following scan parameters: echo time  = 144 ms, repetition time  = 2 s, slice thickness 1.5 cm, in plane resolution 1×1 cm^3^, spectral bandwidth 2000 Hz and 1024 sample points. Signal from skull fat was suppressed by placing 10 saturation bands in a circular pattern over the PRESS box. Total scan time was around 8 min. N-acetylaspartate (NAA) peak areas were analyzed bilaterally in the 12 voxels corresponding to the centrum semiovale, with exclusion of voxels including lesions, and expressed as ratios to Cr, which is assumed to be relatively stable [17] (**Figure 2**).

**Figure 3:**
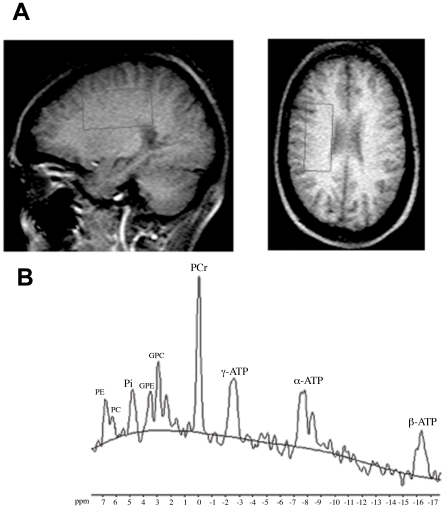
^31^P Magnetic resonance spectroscopy. (**A**) Volume of interest in the centrum semiovale for ^31^P MRS. (**B**) Typical decoupled ^31^P-spectrum with the peaks for PCr, phosphorylethanolamine (PE), phosphorylcholine (PC), inorganic phosphate (Pi), glycerophosphorylethanolamine (GPE), glycerophosphorylcholine (GPC), and the three peaks for ATP.

^31^P MRS was preceded by the acquisition of transverse, sagittal and coronal MRI series in order to position the volume of interest (6×3×3 or 6×2.5×3 cm^3^; 45–54 cc) in the right centrum semiovale (**Figure 3**). The distance between the center of the surface coil and the middle of the volume of interest was always less than 7 cm, to allow for good quality spectra. Scan parameters were: repetition time  = 4.5 s, spectral bandwidth 3000 Hz, 1024 sample points, and 256 measurements were averaged for the decoupled spectra. The volume of interest contained on average 3 (0–9) visible focal lesions in the whole patient group. However, the total lesion volume was very small compared to the total volume of interest, reflecting less than 5% of the total volume. Cortical grey matter contamination in the volume of interest was also below 5%. Total scan time was around 20 min. The processed spectra obtained by ^31^P MRS were curve fitted to provide metabolite peak areas (**Figure 3**). The ^31^P spectra showed peaks of PCr, inorganic phosphate (Pi), the three phosphates of ATP (α- ATP, β-ATP, γ-ATP), phosphorylethanolamine (PE) and phosphorylcholine (PC)  =  phosphomonoesters (PME), glycerophosphorylethanolamine (GPE) and glycerophosphorylcholine (GPC)  =  phosphodiesters (PDE). In the statistical analysis the ratios PCr/β-ATP, PCr/total ^31^P and PCr/Pi were used as measures of the PCr energy status. Total ATP/total ^31^P was also computed and the ratios of PME and PDE to β-ATP were included as well. β-ATP is used as reference for ATP because it does not contain contributions of other compounds known to be present in brain tissue [17]. NAWM was defined as white matter outside the T2*-*hyperintense foci on brain MRI.

### Post-mortem brain tissue

Frozen brain samples were obtained from the Netherlands Brain Bank (Amsterdam, the Netherlands) and the UK Multiple sclerosis tissue bank (London, UK). We investigated NAWM from brain slices from 10 patients who had died with secondary progressive MS (age at death 64±11 years; 5 women and 5 men; postmortem time 9±3 h), and 10 controls without CNS disease (age at death 71±11 years; 5 women and 5 men; postmortem time 8±3 h). Age and postmortem intervals were not significantly different between MS patients and controls p = 0.105; p = 0.596).

### Creatine kinase B

Equal amounts (500 mg) of frozen macroscopically NAWM samples were homogenized with a Teflon-glass homogenizer in ice-cold 50 mM Tris buffer, pH 7.6. Homogenates were centrifuged at 600 x g for 10 min, the pellet was discharged and the supernatant was centrifuged at 8000 x g for 20 min. The supernatant of the second centrifugation was collected for determination of cytosolic CK, which represents CK-B. This separation procedure was used to remove the mitochondrial CK fraction.

CK-B concentrations were assessed using a non-competitive sandwich ELISA. A monoclonal mouse anti-human CK-B, a polyclonal antibody rabbit anti-human CK-B and purified CK-B protein were obtained from Abcam (Cambridge, UK). The anti-human CK-B antibody reacts only with the CK-BB dimer, and not with the B subunit of CK-MB. A 96-well plate was coated with 100 µl per well of monoclonal anti-human CK-B antibody (diluted to 0.5 µg/ml with 0.05 M sodium bicarbonate, pH 9.6) and incubated overnight at 4°C. Wells were washed once with 300 µl Tris buffered saline (20 mM Tris and 150 mM NaCl) containing 0.1% Tris-Buffered Saline Tween-20 using a microplate washer. The plate was then blocked for 1 h with blocking buffer (200 µl per well) consisting of 1% bovine serum albumin made up in Dulbecco's phosphated buffer saline (pH 7.35). The blocked plate was washed once with 300 µl Tris-Buffered Saline Tween-20. CK-B protein standards were made in Dulbecco's phosphated buffer saline at final concentrations of 0.22 µg/ml, 2.2 µg/ml, 22 µg/ml and 220 µg/ml. Standards and cytosolic white matter samples were added (100 µl per well) and the plate was incubated at room temperature for 2 h. Wells were washed 4 times with 300 µl Tris-Buffered Saline Tween-20. Thereafter, each well was incubated with 100 µl of rabbit anti-human CK-B diluted in Dulbecco's phosphated buffer saline (0.5 µg/ml) for 2 h at room temperature. The plate was washed four times with 300 µl Tris-Buffered Saline Tween-20, and after washing each well was incubated with 100 µl polyclonal anti-rabbit-HRP (0.2 µg/ml). The plate was then incubated in the dark at room temperature for 1 h. After washing, 100 µl per well of substrate (1-step Ultra TMB-ELISA, Pierce, Rockford, IL) was added and the plate was incubated for 10 min in the dark. Color change was monitored visually. The reaction was stopped by the addition of 100 µl of 1 N HCl per well. Absorbance at 450 nm was read on a spectrophotometer.

Cytosolic CK activity was assayed using an EnzyChrom™ CK Assay Kit (BioAssay Systems, Hayward, CA). The assay is based on enzyme-coupled reactions in which PCr and asdenosine diphosphate (ADP) is converted to Cr and ATP by CK. The generated ATP is used to phosphorylate glucose by hexokinase to generate glucose-6-phosphate, which is then oxidized by nicotinamide adenine dinucleotide phosphate (NADP) in the presence of glucose-6-phosphate dehydrogenase. The rate of change of NADP absorbance, measured at 340 nm, is proportionate to the CK activity in the sample. Activity measurement was initiated by the addition of PCr. Withholding PCr addition until all the endogenous ATP was consumed in the CK reaction compensated for the presence of endogenous ATP in the samples.

### Statistics

The Kruskal-Wallis test was used to evaluate group differences in MRS with GraphPad Prism version 4.00 for Windows (San Diego, CA). When a statistically significant difference was found, the Dunn test was applied to assess differences. Associations were assessed by Spearman's rank-correlation coefficient. We evaluated the correlation between NAA/Cr and PCr/β-ATP or PCr/total ^31^P by partial correlation testing, controlling for age and sex, by using the statistical package for Social Sciences (SPSS 12.0 for Windows, Chicago, IL). Comparisons between MS subjects and controls for CK assays were performed by means of the Mann–Whitney U test. Results are expressed as means ± standard deviation. All reported p values are two sided.

## Results

### MRS

^1^H MRS in the NAWM of the centrum semiovale showed a difference in NAA/Cr ratio between the three groups (p = 0.02), which was explained by a reduction in the progressive MS group compared to healthy controls (Dunn's post test p<0.05) (**Table 2**). Some decrease was also observed in the benign MS group, but the difference with the healthy controls was not significant.

**Table 2:** Results of the ^1^H and ^31^P MRS measurements.

	Controls n = 10	Benign MS n = 9	Progressive MS n = 17
NAA/Cr	2.19 (0.16)	2.06 (0.22)	2.00 (0.21)#
PCr/β-ATP	1.95 (0.28)	2.19 (0.35)	2.42 (0.64)#
PCr/total ^31^P	0.21 (0.02)	0.23 (0.04)	0.25 (0.04)‡
PCr/Pi	3.43 (0.54)	3.64 (0.88)	3.89 (0.81)
PME/β-ATP	0.66 (0.15)	0.70 (0.20)	0.72 (0.16)
PDE/β-ATP	1.61 (0.23)	1.75 (0.30)	1.55 (0.30)
Total ATP/total ^31^P	0.46 (0.03)	0.455 (0.02)	0.45 (0.03)
β-ATP/total ^31^P	0.11 (0.01)	0.10 (0.01)	0.11 (0.02)

Values are mean ±SD.

PCr, phosphocreatine; Cr, creatine; NAA, N-acetylaspartate; Pi, inorganic phosphate; PME, phosphomonoesters; PDE, phosphodiesters; ATP, adenosine triphosphate; P, phosphorus.

**#**p = 0.02; ‡ p = 0.03 (Kruskal-Wallis, followed by Dunn test).

**Figure 4:**
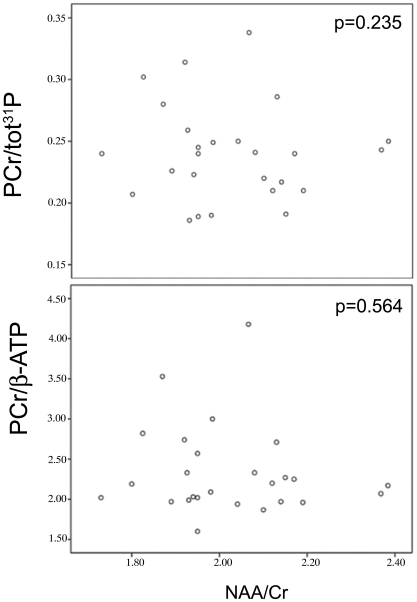
Correlation between NAA/Cr and PCr/total ^31^P and PCr/β-ATP in the MS subjects.

^31^P MRS showed a significant difference among the 3 groups in the PCr/β-ATP ratio (p = 0.02) and PCr/total ^31^P ratio (p = 0.03), which was explained by an increase in the progressive group compared to healthy controls (Dunn's post test p<0.05) (**Table 2**). Compared to the healthy controls, PCr/β-ATP and PCr/total ^31^P ratios were slightly increased in benign MS subjects, but this was not significant. There were no differences between MS subjects and controls with regard to the other metabolite ratios. Considering the whole MS group, there was no correlation between the NAA/Cr ratio and both PCr/β-ATP and PCr/total ^31^P ratios (**Figure 4**).

### Creatine kinase B

**Figure 5:**
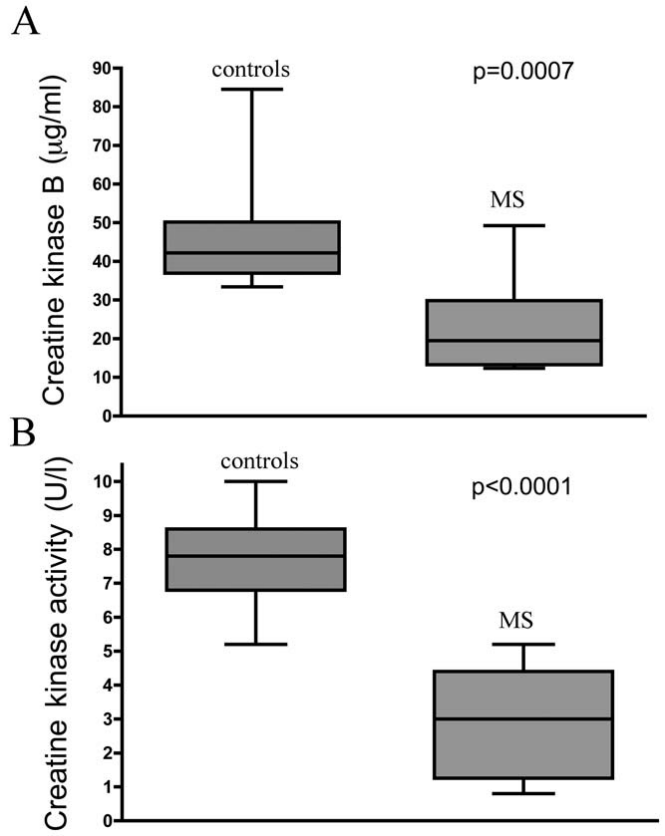
Creatine kinase B. Box plots of (**A**) creatine kinase B levels and (**B**) creatine kinase B activity in the NAWM in the MS subjects and controls.

CK-B levels in the NAWM were lower in the MS subjects (22.41±11.51 µg/ml) than in the controls (46.28±14.60 µg/ml; p = 0.0007). White matter cytosolic CK activity, which corresponds to CK-B activity, was lower in the MS subjects (2.89±1.53 U/l) than in the controls (7.76±1.30 U/l; p<0.0001). Box plots of the CK-B levels and cytosolic CK activity are shown in **Figure 5**. There was a good correlation between CK-B levels and activity (r = 0.650; p = 0.0019).

## Discussion

Our finding of increased PCr/β-ATP and PCr/total ^31^P ratios in the NAWM of subjects with progressive MS is in accordance with two previous studies [15] [16]. Since ^31^P MRS has less spatial resolution than ^1^H MRS, a larger volume of interest is needed, and such a larger volume can contain some focal MS lesions and adjacent cortical grey matter. However, considering the whole MS group, both total lesion volume and cortical grey matter contamination was each less than 5%. Similar to the two previous studies we measured PCr ratios, and not estimated absolute values for PCr, which require the use of calibrated phantoms for each patient. β-ATP/total ^31^P ratios were not different between MS subjects and controls, indicating that the elevated PCr/β-ATP and PCr/total ^31^P ratios in the NAWM in MS reflect elevated PCr levels.

Compared to controls, patients with benign MS showed a trend for elevated PCr ratios in the NAWM but the difference was not significant. NAA is an amino acid produced by mitochondria in neurons, and the NAA/Cr ratio is regarded as a measure for axonal function and integrity. The lack of correlation between the NAA/Cr ratio and PCr/β-ATP and PCr/total ^31^P ratios suggests that the PCr increase in progressive MS is not secondary to axonal dysfunction or loss. Another argument against a secondary phenomenon is that PCr ratios were not elevated in focal MS lesions [16].

Our results suggest that a reduced PCr metabolism in the NAWM in progressive MS is caused by decreased activity of CK-B, which is confined to astrocytes [10] [11] [12]. Unfortunately, it is impossible to analyse PCr in postmortem brain samples, because PCr degradation starts already within a few minutes after death. In rabbit brain studied 1 h after death PCr was no longer measurable [18].

It has been suggested that oxidative stress may reduce CK-B activity [19]. A number of studies found evidence of increased production of reactive oxygen species in inflammatory lesions, cerebrospinal fluid and plasma of subjects with MS [20] [21] [22]. However, loss of CK-B activity by oxidative stress appears to be caused by posttranslational oxidative modification of the enzyme [19]. In our MS samples, the levels of CK-B were significantly reduced. Previous studies have shown that transcription of CK-B in human astroglial cells *in vitro* is induced by cAMP [23] [24]. White matter astrocytes contain β**_2_** adrenergic receptors, which upon activation by norepinephrine increase the levels of cAMP [25]. We have previously reported that, in contrast to other forms of CNS injury, astrocytes in MS cerebral white matter are deficient in β**_2_** adrenergic receptors [25]. A deficiency in astrocytic β**_2_** adrenergic receptors might provide an explanation for the decrease in white matter CK-B levels. However, this relationship is at this moment speculative, and warrants further research.

It is tempting to speculate that a reduced energy metabolism in astrocytic axonal end-feet during increased demands might play a role in the degeneration of oligodendrocytes and axons in MS. The propagation of action potentials along myelinated axons not only leads to expulsion of K^+^ but also to rapid vesicular release of glutamate at the nodes [26]. At the level of the astrocyte endings, glutamate uptake is accomplished by Na^+^-dependent transporters [13] [14], which move glutamate into astrocytes against a steep concentration gradient by coupling glutamate translocation to the transmembrane Na^+^, K^+^ gradients. These gradients are maintained by membrane Na^+^/K^+^-ATPase, such that glutamate uptake is ultimately ATP dependent. Reduced activity of the astrocytic Na^+^/K^+^-ATPase will lead to higher extracellular K^+^ concentrations and may induce reversal of glutamate uptake by glutamate transporters [27]. Support for a role of PCr in astrocyte glutamate uptake has been obtained in experiments showing that a glutamate challenge to cultured astrocytes was associated with enhanced PCr consumption [28] [29].

Glutamate toxicity might be responsible for apoptotic cell death of oligodendrocytes in MS [30] [31]. However, it is likely that additional factors are required to explain why this has a tendency to occur in a focal and not in a more diffuse manner. Glutamate toxicity might provide an explanation for the diffuse axonal degeneration in MS [30] [32]. Central myelinated axons express functional α-amino-3-hydroxyl-5-methyl-4-isoxazole-propionic acid (AMPA) and kainate receptors, which upon overstimulation lead to an increase of intra-axonal Ca^2+^ levels [33] [34]. Abnormally increased Ca^2+^ in axons will activate catabolic enzymes, impair mitochondrial function and axonal transport, and this may lead to axonal degeneration [35]. It has been suggested that glutamate toxicity might be further exaggerated by inflammation, because glutamate is also released in large quantities by activated immune cells [32] [36]. Elevated glutamate levels have been demonstrated throughout the brain NAWM [37], and in the cerebrospinal fluid of subjects with MS [38]. The expression of glutamate transporters in MS white matter is increased, which constitutes a known regulatory response of glial cells to toxic levels of glutamate [39].

In summary, we show reduced CK-B activity in postmortem obtained NAWM of subjects with progressive MS. Our data raise the possibility that this may be responsible for a defective PCr metabolism in astrocytes. We speculate that this abnormality might provide an explanation for glutamate toxicity thought to be responsible for the degeneration of oligodendrocytes and axons in MS. Further *in vivo* studies are required to investigate in more detail the role of PCr metabolism in astrocyte glutamate uptake, as this may provide new insights in the pathogenesis of MS.
